# Characteristics and Carcinogenic Risk of PM_2.5_-Bound Polycyclic Aromatic Hydrocarbons from Charcoal Barbecue (Moo Kratha) Restaurants in Chiang Mai, Thailand

**DOI:** 10.3390/toxics14070643

**Published:** 2026-07-22

**Authors:** Thanawat Komonnithiphong, Kotchakorn Khammoon, Sakaewan Ounjaijean, Kongsak Boonyapranai, Hataichanok Chuljerm, Kanokwan Kulprachakarn, Wiritphon Khiaolaongam, Anurak Wongta, Surat Hongsibsong, Sawaeng Kawichai

**Affiliations:** 1 Research Institute for Health Sciences (RIHES), Chiang Mai University, Chiang Mai 50200, Thailand; thanawat_komo@cmu.ac.th (T.K.); kotchakorn_kh@cmu.ac.th (K.K.); 2 School of Health Sciences Research, Research Institute for Health Sciences (RIHES), Chiang Mai University, Chiang Mai 50200, Thailand; sakaewan.o@cmu.ac.th (S.O.); kongsak.b@cmu.ac.th (K.B.); hataichanok.ch@cmu.ac.th (H.C.); kanokwan.kul@cmu.ac.th (K.K.); wiritphon_k@cmu.ac.th (W.K.); anurak.wongta@cmu.ac.th (A.W.); surat.hongsibsong@cmu.ac.th (S.H.)

**Keywords:** PM_2.5_, polycyclic aromatic hydrocarbons (PAHs), charcoal barbecue (Moo Kratha), benzo[a]pyrene equivalent (TEQ_BaP_), incremental lifetime cancer risk (ILCR)

## Abstract

Barbecue (Moo Kratha) restaurants are prevalent in Chiang Mai City, Thailand. However, their PM_2.5_-bound polycyclic aromatic hydrocarbon (PAH) emissions, compounded by the region’s severe seasonal smoke haze, remain poorly characterized. This study investigated the chemical composition and carcinogenic risk of PM_2.5_-bound PAHs in this present environment. Ten PM_2.5_ samples were collected from ten charcoal Moo Kratha restaurants during peak evening hours in February 2026 (early dry season) using a portable air sampler operated as a fixed-location (area) sampler positioned at the customer dining table (2 L min^−1^, 180 min) on quartz fiber filters, and the 16 US EPA priority PAHs were analyzed by GC-MS. Carcinogenic risk was assessed using benzo[a]pyrene toxicity equivalent (TEQ_BaP_) concentrations and incremental lifetime cancer risk (ILCR) for adult and child customers (diners) via the inhalation pathway. The mean concentrations of PM_2.5_ and total PAHs were 87.64 µg m^−3^ and 132.74 ng m^−3^, respectively, with carcinogenic PAHs contributing 52.3%; the mean TEQBaP of 15.34 ng m^−3^ provides an internal index of the carcinogenic potency of the PAH mixture. The mean inhalation ILCR for a frequent diner was 2.82 × 10^−5^ for adults and 2.12 × 10^−5^ for children, rising to 6.16 × 10^−5^ and 4.62 × 10^−5^, respectively, at the most contaminated site; even an occasional (monthly) diner exceeded the 10^−6^ negligible threshold. These findings indicate that charcoal Moo Kratha restaurants are distinct, persistent sources of carcinogenic PAHs that place diners within the upper part of the tolerable cancer-risk range; worker exposure is expected to be higher but was not quantified here, underlining the need for ventilation and occupational-health strategies.

## 1. Introduction

Fine particulate matter (PM_2.5_) is widely recognized as a severe environmental health hazard, owing to its capacity to penetrate the respiratory tract, deposit in the alveolar region, and deliver toxic chemical constituents into the systemic circulation [1,2]. A substantial body of epidemiological and toxicological evidence consistently links prolonged PM_2.5_ exposure to an elevated incidence of cardiopulmonary disease, oxidative stress, and premature mortality [3]. Among the organic constituents associated with PM_2.5_, polycyclic aromatic hydrocarbon (PAH) semi-volatile organic compounds formed predominantly during the incomplete combustion of biomass, fossil fuels, and other organic matter are of particular concern [4,5]. Because various PAH compounds, particularly benzo[a]pyrene (BaP), are considered probable or demonstrated human carcinogens, this class of chemicals has been studied extensively in environmental risk assessment [6,7,8,9]. In Southeast Asia, PM_2.5_-bound PAH pollution is strongly seasonal and is frequently driven by agricultural residue burning and forest fires. Northern Thailand, and the Chiang Mai basin in particular, is a well-documented hotspot for the recurrent smoke haze episodes that develop during the dry season from January to April [10,11,12,13,14]. Throughout this period, regional biomass burning markedly elevates ambient PM_2.5_ concentrations, often driving them well beyond the limits recommended by the World Health Organization (WHO) and stipulated by Thailand’s National Ambient Air Quality Standards [15,16,17,18]. As the dry season advances and temperature inversions trap air within the basin, PM_2.5_ progressively accumulates from January and February onward, degrading local air quality before reaching its annual maximum in March [3,6,19,20].

Diagnostic ratios, positive matrix factorization (PMF), and carbon isotope analysis have collectively confirmed that biomass burning is the dominant source of carcinogenic PAHs in the regional ambient air during the haze season [21,22,23]. Ambient PM_2.5_ in Chiang Mai City has further been shown to exhibit pronounced oxidative potential, closely associated with 4 and 5 ring PAHs [3], while human biomonitoring studies have detected substantial PAH accumulation in biological matrices such as scalp hair among students sampled during the burning season [24,25,26]. Although these regional outdoor haze dynamics are now well characterized, a notable gap remains concerning the contribution of indoor microenvironmental sources, particularly commercial cooking, superimposed on this already elevated pollution baseline.

Commercial cooking, as well as high-temperature meat charbroiling and grilling, generates substantial concentrations of fine particulate matter and organic aerosols [27,28,29]. The process combines the pyrolysis of animal lipids with the incomplete combustion of charcoal, releasing appreciable quantities of particulate-phase PAHs [27,30,31]. Field measurements in charbroiling restaurants have reported a PM_2.5_-to-PM_10_ ratio of approximately 0.98, implying that virtually all emitted particles are respirable, with PAH concentrations in exhaust reaching at least 4000 ng m^−3^ [32]. Such environments impose disproportionate health burdens: occupational exposure studies indicate that grill workers and kitchen staff incur incremental lifetime cancer risks (ILCRs) exceeding acceptable thresholds because of prolonged inhalation and dermal contact with cooking fumes [30,33]. Field investigations in South Korea, India, and Taiwan have repeatedly reported ΣPAHs concentrations in commercial cooking and PM_2.5_ ranging from tens to hundreds of ng m^−3^, with a high carcinogenic PAH content [32,34,35]. Corresponding ILCR estimates for adult and child customers (diners) exceed the US EPA benchmark of 10^−6^, especially under conditions of inadequate ventilation [34,35,36].

Despite this broad recognition of the hazards posed by cooking emissions, considerable uncertainty persists regarding their significance within the specific context of Chiang Mai City. Charcoal barbecue establishments are ubiquitous across the city, operate year-round, and typically burn charcoal within semi-enclosed spaces. Characterizing the associated occupational and public health risks is especially important here, because emissions from these venues are superimposed on an already heavily contaminated regional background [6,37,38,39]. In the absence of source-resolved data on these microenvironments, regional PM_2.5_ mitigation strategies and occupational health standards cannot be fully informed or effective.

Accordingly, the present study investigates the chemical characteristics and associated health risks of PM_2.5_-bound PAHs emitted by charcoal barbecue (Moo Kratha) restaurants in Chiang Mai City, Thailand. Ten PM_2.5_ samples were collected from ten such restaurants in February 2026, representing the early phase of the dry season. The specific objectives were (1) to quantify the mass concentrations of PM_2.5_ and the 16 US EPA priority PAHs in the indoor/near exhaust environment; (2) to conduct a comprehensive health risk assessment employing toxicity equivalent (TEQ_BaP_) concentrations and incremental lifetime cancer risks (ILCRs) to evaluate the carcinogenic risks to restaurant customers (diners). This study provides localized evidence for urban air quality management and occupational safety guidelines in Chiang Mai City and similar environments by distinguishing the emission source at the start of the haze season.

## 2. Materials and Methods

### 2.1. Sampling Sites

**Figure 1:**
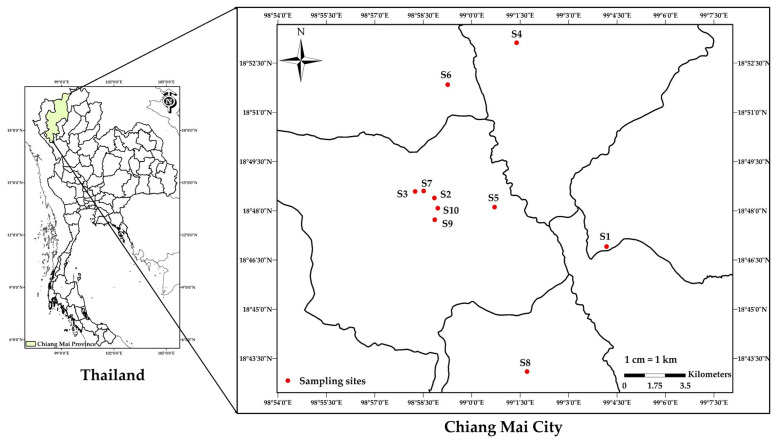
The location of sampling sites in Chiang Mai City.

The study was carried out in Chiang Mai City, located in upper northern Thailand, a significant center for commerce, education, and tourism. Local emissions are increasing because of economic and transportation activities. Traffic emissions, biomass combustion, and cooking-related combustion contribute to seasonal air pollution in Chiang Mai City, Thailand. Ten Moo Kratha restaurants, employing charcoal for grilling meat, were investigated. This cooking process is a potential source of indoor air pollutants, particularly particulate matter and combustion-related byproducts. The selected restaurants were called S1 to S10 and distributed around Chiang Mai City, Thailand (Figure 1). The selected sites differed in terms of restaurant size, seating capacity, structural characteristics, and surrounding environments, such as proximity to major roads or residential areas. These characteristics, summarized for each site in Table S1, are expected to influence local emission strength, dilution, and the resulting indoor PM_2.5_ and PAH concentrations.

The PM_2.5_ sample collection was conducted in February 2026. The measurements often started at nightfall (18:00 to 19:00) and finished in the mid-to-late evening (post 20:00), lasting 3 h, a period characterized by the highest consumer activity at barbecue restaurants and elevated cooking smoke intensity. The sampler was placed on a dining table within the seating area, at approximately the breathing-zone height of seated customers, rather than being worn by any individual; the measurements therefore characterize the area concentrations to which diners are exposed during a meal. The 3 h sampling window corresponds to the typical maximum dining time at these restaurants, where a single Moo Kratha barbecue session is generally limited to about three hours, so that each sample represents a complete customer-visit exposure rather than only a brief high-activity peak. In Moo Kratha restaurants, grilling is performed by the customers themselves at each dining table on an individual charcoal brazier fitted with a domed grill pan; there is no separate enclosed cooking kitchen for the grilling step, so the charcoal-combustion and animal-fat pyrolysis emission source is located at the table, within the seated customer’s breathing zone. The selected sites differed in terms of restaurant size, seating capacity, structural characteristics, and surrounding environments, such as proximity to major roads or residential areas. These factors may influence local traffic intensity and contribute to variations in ambient and indoor particulate matter concentrations.

### 2.2. Sample Collection

PM_2.5_ samples were collected from ten Moo Kratha restaurants using a personal portable air-sampling pump (AirChek 2000, SKC Inc., Eighty Four, PA, USA) operated at a flow rate of 2 L min^−1^ for 180 min. Particulate matter was collected on 37 mm quartz fiber filters (Whatman, Cytiva, Maidstone, UK). Prior to and after sampling, filters were conditioned under controlled temperature and humidity and weighed using a microbalance to determine PM_2.5_ mass concentrations gravimetrically (Mettler Toledo, Greifensee, Switzerland). Airborne particulate concentrations were calculated based on the sampling flow rate and duration. Following sampling, the filters were stored at temperatures below −20 °C until analysis. PAHs associated with PM_2.5_ were subsequently analyzed using GC-MS, following standard extraction and cleanup procedures.

### 2.3. Sample Analysis

Sixteen US EPA priority polycyclic aromatic hydrocarbons (PAHs) were extracted and quantified, namely Naphthalene (Nap), Acenaphthylene (Acy), Acenaphthene (Ace), Fluorene (Flu), Phenanthrene (Phe), Anthracene (Ant), Fluoranthene (Fla), Pyrene (Pyr), Benzo(a)anthracene (BaA), Chrysene (Chr), Benzo(b)fluoranthene (BbF), Benzo(k)fluoranthene (BkF), Benzo(a)pyrene (BaP), Dibenz(a,h)anthracene (DahA), Indeno(l,2,3-cd)pyrene (IcdP) and Benzo(g,h,i)perylene (BghiP). The PAH compounds were analyzed and identified based on their retention time and the qualifier ions of standards in selected ion mode (SIM) using a modified method derived from prior protocols [6]. Each quartz filter was placed into a 20 mL glass vial. Before extraction, internal standards were spiked into the samples for evaluating recovery: 20 µL of Acenaphthene-D_10_ (200 µg L^−1^) for low-molecular-weight compounds and 20 µL of Perylene-D_12_ (200 µg L^−1^) (both Dr. Ehrenstorfer, LGC Standards, Augsburg, Germany) for high-molecular-weight compounds. The quartz filters were put through to ultrasonic extraction three times, using 5 mL of dichloromethane (DCM; J.T. Baker, Phillipsburg, NJ, USA) for 10 min per cycle at a controlled temperature of approximately 10 °C. The extracts were then filtered using a Whatman 0.2 µm PTFE filter (Cytiva, Maidstone, UK) to remove suspended particulates. The filtrate was concentrated using a gentle air stream in a water bath at 32 °C until nearly dry, then reconstituted in 0.2 mL of ethyl acetate (EA) for analysis.

### 2.4. GC-MS Analysis

Instrumental analysis was performed via a gas chromatograph (Agilent 7890A, Agilent Technologies, Palo Alto, CA, USA) coupled with an Agilent 5975C mass spectrometer detector (MSD) (Agilent 7890A, Agilent Technologies, Palo Alto, CA, USA). Separation was carried out using an HP-5MS capillary column (30 m × 0.25 mm × 0.25 µm film thickness). The inlet temperature was set at 280 °C. High-purity helium was used as the carrier gas at a constant rate of 1 mL min^−1^. The thermal gradient protocol started at 65 °C (held for 2 min), increased to 180 °C at a rate of 2 °C min^−1^ (held for 2 min), then increased to 230 °C at 10 °C min^−1^ (held for 3 min). Finally, the temperature was increased to 290 °C at a 10 °C min^−1^ (held for 10 min). The injection was performed in splitless mode at 275 °C. The mass selective detector operated in electron impact (EI) mode at 70 eV, with an ion source temperature of 250 °C. Data were collected in full scan mode (50–350 *m*/*z*) for compound identification through comparison of mass spectra with library data, while SIM was applied for quantification based on retention times and comparison with calibration standards. The data were obtained and analyzed using Agilent Chem-Station software (version E.02.02.1431, 1989–2011, Agilent Technology, Palo Alto, CA, USA). Finally, the analytical procedure was adapted from Kawichai et al. (2020), with minor modifications [6].

### 2.5. Quality Assurance and Quality Control

The repeatability and reproducibility of the PAH analysis were rigorously validated. Instrument calibration was conducted utilizing a multi-point standard solution for each of the 16 US EPA priority PAHs, with coefficients of determination (R^2^) above 0.99 for all analytes, indicating exceptional linearity. The limits of detection (LODs) and limits of quantification (LOQs) ranged from 0.35 to 4.99 µg L^−1^ and 1.17 to 16.63 µg L^−1^, respectively. Analytical accuracy was evaluated using a spiking technique (*n* = 6), with recovery rates for most PAHs consistently falling within the acceptable range of 76.1 to 121.4%. Precision was confirmed with six replicate analyses at a concentration of 5 µg L^−1^, with relative standard deviations (% R.S.D.), typically below 15%, demonstrating the strong reproducibility of the GC-MS method. Each sample batch included blank and parallel samples investigated to confirm the absence of interference or cross contamination. A solvent blank and PAH-16 standard (SV Calibration Mix #5, Catalog No. 31011; Restek Corporation, Bellefonte, PA, USA) were run daily for assessing instrument performance. A solvent blank and PAH-16 standard [40] were run daily for assessing instrument performance. The accuracy and reproducibility of the method were verified as well by analyzing a certified standard reference material (SRM; NIST SRM 1649b urban dust) (National Institute of Standards and Technology, Gaithersburg, MD, USA) under comparable extraction and GC-MS conditions to the samples. The recoveries of the target PAHs according to the certified values ranged from 18.98% (Flu) to 104.57% (Chr), with a mean of 54.88 ± 4.76%, confirming the accuracy of the method for the PAHs reported in this study.

### 2.6. Health Risk Assessment

PAHs can enter the human body by inhalation, ingestion and dermal contact. Because the present study measured PAHs in the airborne PM_2.5_-bound phase, the quantitative cancer-risk assessment reported here is confined to the inhalation pathway, for which an airborne concentration is the dimensionally appropriate input; the ingestion and dermal pathways are discussed qualitatively in Section 3.4. BaP is the principal recognized carcinogenic PAH, classified as Group 1 by the IARC, whereas the other congeners are regarded as probable or possible human carcinogens; the classification of all 16 PAH compounds is summarized in Table 1. The BaP toxic equivalent concentration (TEQ_BaP_) quantifies the carcinogenic potential of PAH compounds by comparing their concentrations to that of benzo[a]pyrene (BaP), a recognized carcinogen. TEQ_BaP_ assigns a toxic equivalency factor (TEF) to each congener relative to BaP; this allows for the assessment of the cumulative risk associated with several carcinogenic PAHs. This study investigated the toxicity of several PAHs in PM_2.5_ samples using TEF-based calculations. It was used to categorize PAHs according to their TEF values and IARC classifications to determine total carcinogenic risk in terms of TEQBaP [41].

Risk assessment must express exposure in the medium sampled. The present study sampled airborne particulate matter, and TEQ_BaP_ is therefore reported in ng m^−3^ [42,43]. Moreover, TEQ_BaP_ concentrations are commonly used for evaluating the risk of carcinogenic efficiency and are associated with the mutagenic capacity of each polycyclic aromatic hydrocarbon (PAH). TEQ_BaP_ was classified as a carcinogenic substance. The TEQ_BaP_ was calculated using the equation [44,45].

(1) $${\mathrm{TEQ}}_{\mathrm{BaP}}=\sum_{i}^{n=1}\left({\mathrm{PAH}}_{i}\;\times \;{\mathrm{TEF}}_{i}\right)$$

$PAH_{i}$ is the concentration of each PAH compound, while $TEF_{i}$ is the toxic equivalency factor [45]. Because restaurant patrons are exposed intermittently rather than continuously, TEQ_BaP_ was first converted to a lifetime-average exposure concentration (LAEC); the incremental lifetime cancer risk from inhalation (ILCRinh) was then obtained using the inhalation unit-risk (UR) approach recommended by the WHO for PAH mixtures in indoor air [46]. Equations (2) and (3) define this calculation.

LAEC = TEQ_BaP_ × (ET/24) × (EF/365) × (ED/LT) × ADAF (2)

ILCRinh = LAEC × UR(3)

where LAEC is the lifetime-average exposure concentration (ng m^−3^); TEQ_BaP_ is the BaP toxic equivalent concentration (ng m^−3^); ET is the exposure time per visit (h); EF is the exposure frequency (visits year^−1^); ED is the exposure duration (years); LT is the averaging lifetime (70 years); ADAF is the age-dependent adjustment factor; and UR is the inhalation unit risk. Sampling covered the full three-hour duration of a Moo Kratha meal, so ET was set to 3 h. An ADAF of 3 was applied to the child receptor, consistent with US EPA guidance for carcinogens acting by a mutagenic mode of action, which BaP is one [47]; ADAF = 1 for adults. Two unit-risk values were applied, and both are reported. The primary basis is the WHO inhalation unit risk for PAH mixtures, 8.7 × 10^−5^ (ng m^−3^)^−1^ expressed per unit BaP-equivalent concentration, which the WHO established as the guideline basis for PAHs in indoor air [46]. As an independent cross-check, the US EPA IRIS inhalation unit risk for BaP (adult basis, 6 × 10^−4^ (µg m^−3^)^−1^) was also applied [48]. Because the visit frequency of a restaurant patron is not well constrained, ILCRinh was evaluated under three scenarios: an occasional diner (12 visits year^−1^), a regular diner (52 visits year^−1^), and a frequent diner (180 visits year^−1^), the last being retained as a conservative upper bound. Exposure duration was taken as 6 years for children and 24 years for adults [49]. All parameters are listed in Table 2. Risk thresholds are the following: <10^−6^ (negligible), 10^−6^ to 10^−4^ (acceptable), and >10^−4^ (high priority) [50,51,52].

Restricting the quantitative cancer-risk assessment to inhalation is consistent with established practice for airborne PM_2.5_-bound PAHs. Subair et al. [8], cited above, state explicitly that the inhalation of airborne particles was the pathway considered in their health risk assessment and combine the airborne concentration (ng m^−3^) with an inhalation rate (m^3^ h^−1^) and a daily exposure time (h day^−1^). Room et al. [53] likewise confine the incremental lifetime cancer risk of PM_2.5_-bound PAHs to inhalation, weighting the benzo[a]pyrene toxic equivalent concentration by an inhalation unit risk, as adopted here. By contrast, the ingestion and dermal equations of the US EPA framework require a benzo[a]pyrene-equivalent concentration in a solid matrix (mg kg^−1^), together with an incidental soil or dust ingestion rate (mg day^−1^) and a soil-to-skin adherence factor (mg cm^−2^); they are therefore not applicable to an airborne concentration.

**Table 2:** Parameters used for the inhalation incremental lifetime carcinogenic risk (ILCR) assessment.

Exposure Variable	Unit	Children	Adult	Reference
Exposure time per visit (ET)	h	3	3	This study
Exposure frequency (EF)—occasional	visits year^−1^	12	12	This study
Exposure frequency (EF)—regular	visits year^−1^	52	52	This study
Exposure frequency (EF)—frequent	visits year^−1^	180	180	[54]
Exposure duration (ED)	year	6	24	[49]
Averaging lifetime (LT)	year	70	70	[55]
Age-dependent adjustment factor (ADAF)	unitless	3	1	[47]
Inhalation unit risk—WHO (primary)	(ng m^−3^)^−1^	8.7 × 10^−5^	8.7 × 10^−5^	[46]
Inhalation unit risk—US EPA IRIS (cross-check)	(µg m^−3^)^−1^	6 × 10^−4^	6 × 10^−4^	[48]

## 3. Results and Discussion

### 3.1. PM_2.5_ Mass Concentrations

**Figure 2:**
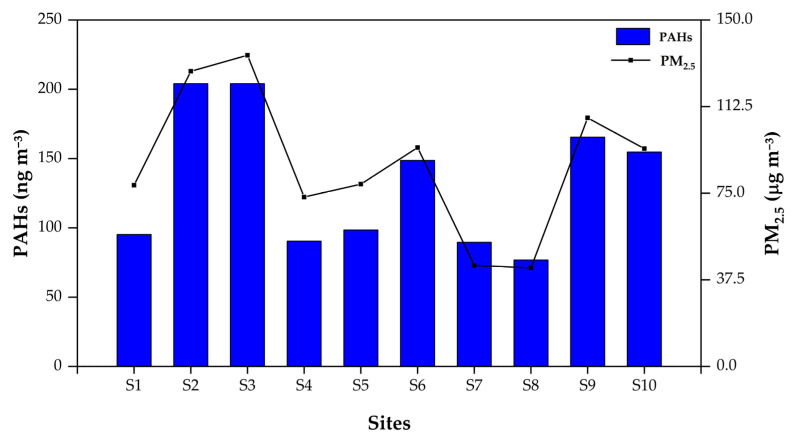
Concentrations of PM_2.5_ (µg m^−3^) and total PAHs (ng m^−3^) in ten Moo Kratha restaurants (S1–S10) in Chiang Mai City, Thailand.

A statistical investigation of PM_2.5_ in the indoor and near-exhaust microenvironments of 10 selected charcoal barbecue restaurants (Moo Kratha) in Chiang Mai City, Thailand, demonstrated an exceedingly concentrated, albeit regionally varied, pollution profile. Continual sampling during high operational hours in February 2026 was one part of this environmental monitoring effort. The results (Table 3) indicate the significant influence of localized solid biomass combustion and meat pyrolysis on the quality of indoor air. The mean PM_2.5_ concentration in the indoor/near exhaust areas was 87.64 ± 30.98 µg m^−3^, with the lowest concentration of 42.67 µg m^−3^ and the highest concentration of 134.72 µg m^−3^. The mean value is significantly higher than the 24 h ambient air quality guideline of 15 µg m^−3^ established by the World Health Organization (WHO) and the NAAQS of 37.5 µg m^−3^ in Thailand, indicating severe problems with indoor air quality in these commercial environments during the early dry season. These exceedances are consistent with previous studies regarding global charcoal combustion cooking environments, which consistently indicate that charcoal grilling produces PM_2.5_ concentrations significantly exceeding ambient outdoor standards due to the incomplete combustion of charcoal fuel and the pyrolysis of animal lipids at a higher temperature [27,31]. The highest PM_2.5_ concentrations were found at sites S3 (134.72 µg m^−3^) and S2 (127.84 µg m^−3^), probably due to the variation in restaurant size, ventilation, seating capacity, distance from main roads, and grilling intensity. The minimum concentrations were found in S8 (42.67 µg m^−3^) and S7 (43.75 µg m^−3^), which indicates substantial variation in indoor air quality among typical restaurants. The substantial inter-site variability in PM_2.5_ and total PAH concentrations is illustrated in Figure 2.

This distinction might be due to changes in ventilation capacity, the amount of cooking, the type of grill, and the layout of the restaurant. In relation to this worldwide health guideline, the environmental reality of these Chiang Mai barbecue-related businesses is completely different. Even the lowest recorded concentration, 42.67 µg m^−3^ at site S8, still exceeded the ambient guideline, despite this venue’s open-air layout and the high degree of natural atmospheric dilution it affords. The significant difference between the lowest concentration at site S8 and the highest at site S3 provides critical insight into the fluid dynamics and aerosol processes influencing indoor microenvironments. Site S8, characterized by an open-air design with unobstructed wind flow, demonstrates the mitigating power of natural ventilation. However, the persistence of an initial level over 40 µg m^−3^ underscores the continuous, high-volume-emission kinetics of charcoal combustion. Conversely, sites S2 and S3 represent worst-case exposure scenarios: semi-enclosed structures where the density of active charcoal burners exceeds the total volume air exchange rate. In these semi-enclosed environments, aerosols rapidly accumulate, disperse, and reach steady-state saturation concentrations that significantly affect human respiratory health.

The present results are compared with regional studies conducted under culturally and technologically comparable conditions. The 2023 study by Vanphanom et al. [56], which evaluated the occupational exposure of grill workers to air pollution from charcoal combustion in Vientiane Capital, Lao PDR, including restaurants and street-food markets, revealed an average PM_2.5_ concentration of 84.8 µg m^−3^. The mean concentration in this study (87.64 µg m^−3^) was similar to that reported for Vientiane (84.8 µg m^−3^); however, because the measurement conditions of that study are not directly comparable and the PAH profiles reported across Southeast Asian studies differ substantially, this similarity in bulk PM_2.5_ should not be taken to imply a uniform or universal exposure and cancer-risk profile across the region [56]. According to Kim et al., the PM_2.5_-to-PM_10_ ratio in urban charbroiling restaurants is approximately 0.98, indicating that most released particles are respirable fine particles. PM_2.5_ concentrations often exceed 100 µg m^−3^ during peak operating hours [32]. Similarly, Li et al. (2014) demonstrated that PM_2.5_ from commercial-grade charbroiling operations was sufficient to trigger significant oxidative stress and inflammatory responses in human bronchial epithelial cells at concentrations comparable to those observed in the present study [30].

Finally, the PM_2.5_ concentrations obtained from this study should be interpreted within an understanding of the early dry season in Chiang Mai City (January–February 2026). In upper northern Thailand, lowered precipitation, reduced wind speeds, and temperature inversions limit the dispersion of regional air pollutants within the Chiang Mai valley basin and enhance the accumulation of biomass combustion aerosol [6,10,11,19]. Although the regional haze typically peaks in March, ambient PM_2.5_ in Chiang Mai City is already elevated above annual averages by January–February [3,17]. The indoor cooking emissions reported here were therefore measured against an already elevated ambient background; the present design does not allow the relative contributions of indoor cooking and outdoor sources to be separated, which should be borne in mind when interpreting the absolute concentrations.

### 3.2. PM_2.5_-Bound PAH Concentrations and Profiles

Table 3 presents the mean concentrations of the 16 US EPA priority PAHs associated with PM_2.5_ at the ten study sites; the corresponding concentrations at each individual site are provided in Table S2. The mean total ΣPAHs concentration was 132.74 ng m^−3^ and ranged from 76.84 to 204.07 ng m^−3^. The total concentration of PAHs was 69.40 ng m^−3^ (52.3% of ΣPAHs) for carcinogenic PAHs and 63.34 ng m^−3^ (47.7%) for non-carcinogenic PAHs (ncPAHs). The predominance of cPAHs indicates that the majority of the PAH burden in the particulate phase in Moo Kratha restaurant environments consists of compounds recognized or believed to be carcinogenic, indicating a potential public health concern for restaurant customers and workers exposed during meal service.

**Figure 3:**
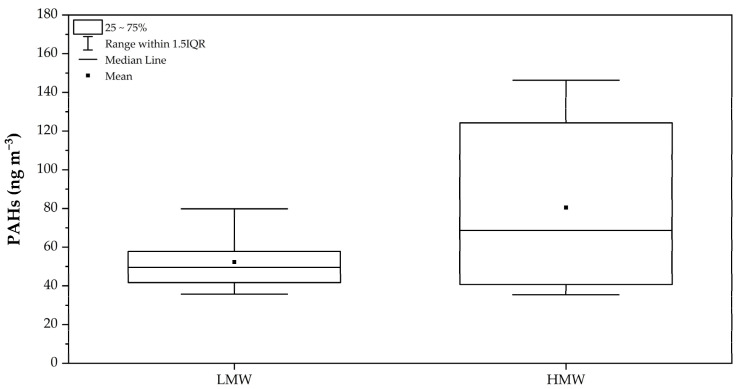
Boxplot comparison of LMW and HMW PAH concentrations (ng m^−3^) in PM_2.5_ samples in ten Moo Kratha restaurants (S1–S10) in Chiang Mai City, Thailand. The box represents the interquartile range (IQR, 25th–75th percentiles), the horizontal line inside the box indicates the median, the solid square denotes the mean, and whiskers extend to values within 1.5 × IQR.

Nap was the predominant PAH precursor, with a mean concentration of 36.20 ng m^−3^ (mean ± SD: 36.20 ± 2.14 ng m^−3^, range: 33.06–39.75 ng m^−3^). The predominance of Nap is explained by its semi-volatile physical and chemical properties, which arise from its lower molecular weight and vapor pressure compared to the EPA 16 PAHs, which therefore favor dispersal to the particle phase in the temperatures that are typical of charcoal-combustion microenvironments [27,41]. Among high-molecular-weight (HMW) PAHs, BghiP and IcdP were detected only intermittently across the ten sites, with mean concentrations of 8.98 ± 9.48 ng m^−3^ and 7.12 ± 9.22 ng m^−3^, respectively. As shown in Figure 3, HMW PAHs displayed both a wider interquartile range (IQR) and a higher median concentration than their LMW counterparts, pointing to greater variability in HMW emissions across the ten restaurants. Such variability is consistent with the marked sensitivity of HMW PAHs to local combustion conditions, including charcoal type, grilling temperature, and the efficiency of fat pyrolysis, all of which differ appreciably between establishments [57]. By contrast, the narrower distribution of LMW PAHs, coupled with their higher mean concentrations, suggests that these lighter, semi volatile congeners are emitted more uniformly and partition partly into the gas phase, allowing them to disperse irrespective of differences in local ventilation [58]. The simultaneous abundance of two- and three-ring LMW species and prominent five- and six-ring HMW congeners (BbF, BghiP, BkF and IcdP) is characteristic of mixed combustion, in which the volatilization of lighter PAHs accompanies the high-temperature pyrolysis of charcoal and animal fat [57]. This profile departs from that reported for Korean charbroiling exhaust, where the three-ring phenanthrene predominated [32]; such discrepancies are generally ascribed to differences in fuel, meat fat content, marinades, and grilling temperature, as well as to the gas particle partitioning of the more volatile generators [57,58]. Regardless of these inter-study differences, the strong representation of HMW PAHs evident in Figure 3, as a group with elevated medians and recurrent outliers, remains of particular toxicological concern, since these compounds are predominantly particle-bound, penetrate deep into the alveolar region, and include the most carcinogenic members of the PAH family, such as BaP, DahA, and IcdP [58]. Notably, IcdP was also among the most abundant carcinogenic congeners reported for ambient PM_2.5_ during Chiang Mai haze episodes [3], suggesting partial overlap between the HMW fingerprint of charcoal cooking and that of the regional biomass burning background.

Chr and BaA were also considered to be most abundant 4-ring carcinogenic PAHs with a mean value of 15.73 ± 11.74 ng m^−3^ (range: 2.56–32.56 ng m^−3^) and 15.66 ± 5.59 ng m^−3^ (range: 9.92–27.35 ng m^−3^), respectively. The high standard deviation for Chr implies significant site-to-site variability, which could be due to differences in fuel type and grilling temperature. BbF (11.70 ± 2.22 ng m^−3^), DahA (2.27 ± 5.70 ng m^−3^) and BkF 8.44 ± 3.42 ng m^−3^ were important contributors to 5-ring compounds, with the distinctive high-temperature combustion PAH signature of charcoal grilling. The contribution of low-molecular-weight 3-ring PAHs Acy (6.47 ng m^−3^), Phe (1.00 ng m^−3^), Ace (2.95 ng m^−3^), Flu (2.59 ng m^−3^) and Ant (0.68 ng m^−3^) was lower, but they were nonetheless present at all sites. The emphasis should be placed on BaP, the only PAH that the IARC has identified as a confirmed human carcinogen (Group 1). The mean BaP concentration in the ten study sites was 8.47 ± 2.14 ng m^−3^ (range: 6.98–14.34 ng m^−3^), which was higher than the WHO standard concentration of BaP in ambient air of 1 ng m^−3^ by a factor of ~8.5. The lowest BaP value (6.98 ng m^−3^) was nearly seven times higher than the WHO guidelines, while the maximum value of 14.34 ng m^−3^ indicates a 14.3-fold exceedance of the standard. Because these measurements represent short-term, peak-service concentrations rather than annual means, this comparison indicates markedly elevated short-term levels rather than an exceedance of the annual mean guideline. These results are in accordance with, and significantly higher than, PAH levels reported in previous studies of charcoal grilling environments. Kim et al. (2020) established that BaP and other carcinogenic PAHs in the exhaust emissions of Korean urban charbroiling restaurants exceeded the WHO BaP guideline [32]. Badyda et al. (2022) demonstrated that BaP levels from charcoal-fueled barbecuing in Poland were substantially higher than the 1 ng m^−3^ threshold in near-source breathing zones [59]. Oliveira et al. (2020) reported higher urine PAH metabolites among grill workers occupationally exposed to environments with BaP exceedances, which was comparable to the present study, giving biomarker level support to the health implications of such indoor BaP concentrations [60]. The persistent BaP exceedance in all ten Moo Kratha restaurants, independent of restaurant size and ventilation arrangement, highlights the need for targeted indoor airquality interventions in this food service environment.

The mean concentrations of ΣPAHs in this investigation were 132.74 ng m^−3^, comparable to, or even higher than, those reported for similar cooking conditions in Asia and other regions throughout the world. Si et al. (2023) measured the concentrations of PAHs in PM_1.0_ in enclosed cooking areas in China during cooking, which ranged from 80 to 200 ng m^−3^, and was comparable to the mean value of our present study [43]. Wei See et al. (2006) [33] quantified PM_2.5_-bound PAHs in commercial Chinese, Malay and Indian food stalls in Singapore, where the highest ΣPAHs occurred at the deep frying-dominated Malay stall (609 ng m^−3^), exceeding the Chinese (141 ng m^−3^) and Indian (38 ng m^−3^) stalls and underscoring that high oil temperature cooking is a strong determinant of PAH emission. For charcoal grilling specifically, Kim et al. (2020) [32] found that PM_2.5_-bound PAHs from urban charbroiling restaurants greatly exceeded those from non-charcoal cooking, and Badyda et al. (2022) reported ILCRs consistently above 10^−4^ for charcoal barbecuing, confirming the high PAH emission intensity of charcoal combustion in commercial cooking [59]. These findings indicate that the Moo Kratha charcoal barbecue microenvironment ranks among the highest indoor PAH levels reported in the peer-reviewed literature, consistent with the combined contributions of charcoal combustion and meat-lipid pyrolysis.

The total particulate-bound PAH concentration averaged at 132.74 ± 48.69 ng m^−3^, spanning 76.84–204.07 ng m^−3^ across the ten restaurants. This range falls squarely within the 61.10–403.80 ng m^−3^ interval reported for PM_2.5_-bound PAHs emitted by eleven cooking styles in Handan, China [36], confirming that the present values are representative of real-world commercial cooking exposures rather than anomalous. The breathing-zone ΣPAH levels measured here are appreciably higher than those typical of gas- or oil-based cooking, a contrast consistent with the comparatively greater PAH yield of charcoal combustion. However, the present values are one to two orders of magnitude lower than undiluted source measurements: ΣPAHs reached 4127 ng m^−3^ in restaurant flue gas exhaust [32]. Charcoal- and briquette-fueled grilling has been demonstrated to emit Σ16 PAHs at levels significantly higher than gas grilling, resulting in incremental lifetime cancer risks that are in excess of the 10^−4^ threshold for both workers and consumers [59]. The intermediate magnitude observed in the present study is expected, because our samples characterize the diluted air actually inhaled by workers and customers some distance from the grill rather than the concentrated exhaust at the emission point. Charcoal pyrolysis, combined with the thermal degradation of animal fat dripping onto hot embers, is the principal mechanism generating these particle-bound PAHs [27,30,31].

From a public health perspective, a notable finding is the substantial carcinogenic PAH burden. Carcinogenic PAHs (cPAHs) averaged 69.40 ng m^−3^ and accounted for 52.3% of ΣPAHs, marginally exceeding the non-carcinogenic fraction (ncPAHs, 63.34 ng m^−3^ 47.7%). BaP is the only PAH classified by the IARC as a Group 1 human carcinogen and the reference compound for toxic equivalency calculations [45], with a mean of 8.47 ng m^−3^, and reached 14.34 ng m^−3^ at the most contaminated site. These values exceed the WHO/European Union reference level of 1 ng m^−3^ by roughly 8.5-fold on average and up to 14-fold at maximum. The contrast with the regional ambient background is marked: during Chiang Mai Province haze episodes, particle-bound BaP averaged at only 0.87 ± 0.42 ng m^−3^ and consistently remained below the 1 ng m^−3^ acceptable level [3], such that the charcoal barbecue microenvironment investigated here carried on the order of ten times the BaP burden of ambient dry season air. The combination of a high cPAH fraction, BaP concentrations consistently above guideline levels, and the deep lung deposition expected for these fine, particle-bound species indicates a considerable inhalation-related carcinogenic potential for both restaurant workers and customers. These results provided the quantitative basis for the toxicity equivalent (TEQ) and incremental lifetime cancer risk (ILCR) assessment presented in Section 3.3.

### 3.3. Health Risk Assessments

The carcinogenic potency of the PM_2.5_-bound PAH mixture was first expressed as the benzo[a]pyrene toxicity equivalent concentration (TEQ_BaP_), obtained by weighting each congener by its toxic equivalency factor relative to BaP [45]. Across the ten Moo Kratha restaurants, the mean TEQ_BaP_ was 15.34 ng m^−3^ (median 12.76 ng m^−3^) and ranged from 9.90 ng m^−3^ at the least contaminated site to 33.51 ng m^−3^ at the most contaminated one (Table 4). These values are reported as an internal index of the carcinogenic potency of the particle-bound PAH mixture and are not benchmarked against the 1 ng m^−3^ annual mean guideline, which applies to BaP alone. The elevated potency is a direct consequence of the carcinogenic PAH dominance described in Section 3.2, in which cPAHs accounted for 52.3% of ΣPAHs and BaP alone averaged at 8.47 ng m^−3^. This potency is particularly notable when compared to the regional dry season background: during Chiang Mai haze episodes, particle-bound BaP averaged at only 0.87 ng m^−3^ and consistently remained below the 1 ng m^−3^ guideline [3]. The charcoal barbecue microenvironment, therefore, concentrates TEQ_BaP_ carcinogenic potency to roughly 18 times the level recorded in the already polluted ambient haze atmosphere, identifying these venues as discrete, high-intensity point sources of carcinogenic PAHs superimposed on a regionally contaminated airshed. To illustrate this potency in terms relevant to the general population, inhalation ILCRs were estimated for adult and child restaurant customers (diners) (Table 4). Because the samples were collected at the dining table, these estimates represent the exposure of restaurant customers (diners), and the adult and child cases denote adult and child diners. In line with the US EPA framework, the child case represents the incremental lifetime cancer risk attributable to exposure occurring during childhood (using a child-specific exposure duration and an age-dependent adjustment factor), not the probability of developing cancer during childhood. Restaurant workers, who are closer to the grills and exposed for longer periods and more frequently, would be expected to experience higher risks; their occupational exposure was not separately quantified in this study and is identified as a priority for future work.

Table 4 presents ILCRinh for the three visit-frequency scenarios. Under the frequent-diner scenario (180 visits year^−1^), retained as a conservative upper bound, the mean ILCRinh was 2.82 × 10^−5^ for adult diners and 2.12 × 10^−5^ for child diners, rising to 6.16 × 10^−5^ and 4.62 × 10^−5^, respectively, at the most contaminated site. Under the regular-diner scenario (52 visits year^−1^, approximately weekly), the corresponding mean values were 8.15 × 10^−6^ and 6.11 × 10^−6^, and under the occasional-diner scenario (12 visits year^−1^, approximately monthly), they were 1.88 × 10^−6^ and 1.41 × 10^−6^. Evaluated according to the accepted risk categories, insignificant below 10^−6^, tolerated or potentially carcinogenic between 10^−6^ and 10^−4^, and of high priority above 10^−4^ [50,51,52], every scenario examined lies above the negligible threshold, and the frequent-diner estimates at the most poorly ventilated, high-throughput locations (sites S2 and S3) approach 10^−4^. The conclusion is therefore robust to the choice of visit frequency: a single three-hour Moo Kratha meal contributes measurably to lifetime cancer risk, and habitual patronage places diners toward the upper end of the tolerable band.

Two factors of these estimates require highlighting. The WHO unit risk is calculated based on lung cancer incidence among coke-oven workers and is designed specifically to be health-protective. The US EPA IRIS inhalation unit risk for BaP (adult basis, 6 × 10^−4^ (µg m^−3^)^−1^) is approximately 145-fold lower, leading to a mean adult risk of 1.95 × 10^−7^ in the frequent-diner scenario (Table 4). The WHO value is utilized as the primary reference since it is the guideline specifically formulated for PAH mixtures in indoor air, the exposure environment of this study, and it aligns with the derivation of the 1 ng m^−3^ BaP reference level employed elsewhere in this paper; the IRIS value is reported as a lower bound so that the divergence between the two authorities is visible rather than concealed. The approximately two orders of magnitude variation is a relevant characteristic of the present toxicological evidence for airborne PAHs, and any single point estimate should be considered with this uncertainty acknowledged. In addition, the relatively modest absolute size of the inhalation risk in relation to the recorded concentrations indicates the intermittent nature of diner exposure three hours per visit compared to a 70-year average lifespan, rather than a low inherent danger of the studied atmosphere. The heightened concentrations of PM_2.5_, BaP, and TEQ_BaP_ noted in Section 3.2 indicate a considerable short-term inhalation burden, and the anticipated deep-lung deposition of mostly particle-bound high-molecular-weight congeners retains toxicological significance.

In this framework, children demonstrate a lower absolute risk than adults, despite the age-dependent adjustment factor of 3, mostly due to their shorter exposure duration (6 years compared to 24 years); but, on exposure basis per year, the risk for children is higher than that for adults. In this commercial information, children are short-term customers rather than permanent residents, causing the typical assumptions regarding exposure frequency and duration conservative in their application; however, family visits to charcoal barbecue restaurants continue to represent an important exposure scenario. The adult ILCR shown above is particular to adult diners. In contrast, restaurant workers are positioned nearer to the braziers work whole shifts rather than only the duration of a single meal, and they are present on most working days; hence, their risk is supposed to significantly exceed the estimations based on diners. The direct quantification of worker exposure through full-shift personal monitoring is a significant direction for future work.

Aligning these values within an international framework indicates that the Moo Kratha environment resides at the higher end of the cooking microenvironment risk level. The range of ΣPAHs observed in the present study is comparable to values reported by Zhang et al. [36] for multiple cooking styles in Handan, China, where PM_2.5_-bound PAHs ranged from 61.10 to 403.80 ng m^−3^, accompanied by estimated adult ILCR values of 1.23 × 10^−6^ to 3.70 × 10^−6^. In comparison, the inhalation ILCR values derived from the present near-source assessment are of the same order of magnitude, consistent with the characterization of breathing-zone concentrations in a close-proximity grilling environment. The present highest value corresponds to charcoal-specific grilling evaluations. Badyda et al. [59] reported ILCRs consistently exceeding 10^−4^ for charcoal and briquette grilling across all exposure scenarios, with the highest values observed for professional cooks. Wu et al. [35] found that 82% (18 of 22) of employees in three commercial cooking businesses in Taiwan had an ILCR higher than that of 10^−5^; in this environment, aldehyde compounds predominated cancer risk (74.9–99.7%) rather than PAHs. Meanwhile, Wei See et al. [33] reported increased levels of particulate phase PAHs in commercial cooking environments when employing various ethnic cooking processes, suggesting that high-temperature cooking techniques significantly enhance occupational PAH exposure. The most important public health observation is the contrast between the indoor charcoal barbecue microenvironment and the ambient environment. The mean TEQ_BaP_ of 15.34 ng m^−3^ and inhalation ILCR of the order of 10^−5^ reported in this study exceed the carcinogenic level of the regional dry season haze, which averaged 0.87 ng m^−3^ particle-bound BaP and ambient ΣPAHs of a few ng m^−3^ [3,6]. In addition to a contaminated seasonal airshed, these microenvironments represent a concentrated occupational and public health risk. Oliveira et al. [60] observed high levels of urinary PAH metabolites in grill workers exposed to comparable BaP levels, and Li et al. [30] showed that charbroiling-derived PM_2.5_ at concentrations like those reported here causes oxidative stress and inflammatory responses in human respiratory cells. The toxicity equivalent and ILCR results recommend targeted interventions engineering local exhaust ventilation and vapor removal, improved cross ventilation in semi-enclosed layouts, worker rotation, and skin-protective and specific inclusion of commercial charcoal cooking in urban air quality and occupational health frameworks. The assessment employs ten cross-sectional samples from the early dry season (February 2026); breathing-zone rather than personal (individually worn) sampling was employed. The standardized U.S. EPA exposure parameters may not fully represent the behavioral patterns of restaurant workers. Additionally, gas-phase PAHs, aldehydes, and other co-emitted volatile organics were not quantified, suggesting that the total carcinogenic health risk may be underestimated. This study presents a toxicity equivalent and incremental lifetime cancer risk assessment of PM_2.5_-bound PAHs from charcoal Moo Kratha barbecue establishments in Chiang Mai City to provide a quantitative evidence base for air quality management and occupational protection strategies.

### 3.4. Strengths and Limitations

Several limitations should be considered when interpreting these results. First, the sampling design comprised ten restaurants with a single 3 h sample collected from each site during the peak evening service period; this window captures the highest-activity, highest-emission stage of restaurant operation but does not represent the full daily or seasonal exposure profile, and the limited number of sites and samples constrains the assessment of temporal variability and spatial representativeness. The ten venues were selected to reflect typical charcoal Moo Kratha establishments distributed across Chiang Mai City but cannot be regarded as a statistically representative sample of all such restaurants. Second, sampling was conducted during February (early dry season), when regional biomass burning and stagnant meteorology elevate the ambient background, so the reported concentrations may differ from those in other seasons. Third, because only one sample was obtained per site, the data are summarized descriptively, and the apparent between-site differences should be regarded as indicative rather than statistically established. Fourth, the incremental lifetime cancer risk was estimated using standard, population-level exposure parameters from the US EPA Exposure Factors Handbook rather than restaurant-specific or individually measured values; in particular, the assumed exposure time may not reflect the actual number of hours per day that individual workers and customers spend on-site, so the risk estimates should be regarded as screening-level approximations of potential risk rather than precise individual risk values. Future work should employ larger numbers of sites, repeated and multi-season sampling, full-shift personal monitoring, and site-specific exposure parameters to refine these estimates.

Fifth, the quantitative cancer-risk estimate provided throughout, is limited to inhalation exposure. Two other mechanisms are possible in the present setting but cannot be derived from airborne concentration and were not quantified. PAHs are generated in meat during grilling, and the consumption of grilled food is a recognized pathway for PAH exposure; quantification requires the assessment of PAHs in cooked food rather than in the air, which was not within the criteria of this study. PAH-laden cooking aerosol is also attached to exposed skin, and dermal absorption may influence the overall dose; quantifying this contribution requires the use of a particle-deposition model (including deposition velocity, exposed surface area, and dermal absorption of the deposited mass) instead of the soil to skin adherence factors utilized for soil and settled-dust risk assessments. A scoping calculation employing a deposition velocity of 0.05 cm s^−1^ suggests a deposited particulate mass of approximately 0.3 mg on an adult’s exposed skin during a three-hour meal, resulting in an ILCR of approximately 10^−7^ small relative to the inhalation estimate but nevertheless subject to at least an order-of-magnitude uncertainty in the deposition velocity. The total risk to diners may consequently be slightly undervalued. Priorities for future research include the direct measurement of PAHs in grilled food and the assessment of PAH-laden particulates deposited on the skin.

## 4. Conclusions

This study demonstrates that charcoal-fueled Moo Kratha restaurants in Chiang Mai City generate substantial concentrations of PM_2.5_ and associated EPA-16 PAHs in their indoor/near exhaust microenvironments, with mean PM_2.5_ (87.6 µg m^−3^) and mean total PAHs (132.7 ng m^−3^) that exceed common ambient guidance levels and include a high fraction of carcinogenic congeners (≈52%). The PAH profile combines abundant low-molecular-weight species with elevated four- to six-ring high-molecular-weight congeners (notably BaA, Chr, BbF, BaP, and IcdP), indicating mixed combustion from charcoal pyrolysis and meat lipid degradation and producing a carcinogenic potency (mean TEQ_BaP_ ≈ 15.3 ng m^−3^), used here as a relative index of mixture toxicity rather than benchmarked against the BaP guideline. The health risk assessment concentrated on the inhalation pathway reveals that the incremental lifetime cancer risks for both adult and child diners remain within the acceptable range (10^−6^ to 10^−4^) across all visit-frequency scenarios analyzed, exceed the negligible threshold even with infrequent visits, as well as close the 10^−4^ priority threshold at the most poorly ventilated sites, indicating a significant public health concern. Given these findings, mitigation should prioritize engineering controls (improved ventilation and localized exhaust), administrative measures (reduced worker exposure durations and worker rotation), and personal protection where appropriate; additionally, urban air quality management and occupational health frameworks should explicitly incorporate emissions from commercial charcoal cooking when assessing local source contributions. Future work should quantify personal exposures across full work shifts, evaluate effectiveness of specific ventilation and exhaust designs in reducing TEQ_BaP_, and explore biomonitoring of exposed workers to link environmental measurements with internal doses.

## Figures and Tables

**Table 1:** Classification of 16 preferred control PAHs.

PAHs	Abbreviation	Ring	TEF	Carcinogenic
Naphthalene	Nap	2	0.001	2B
Acenaphthylene	Acy	3	0.001	3
Acenaphthene	Ace	3	0.001	3
Fluorene	Flu	3	0.001	3
Phenanthrene	Phe	3	0.001	3
Anthracene	Ant	3	0.01	3
Fluoranthene	Fla	4	0.001	3
Pyrene	Pyr	4	0.001	3
Benzo[a]anthracene	BaA	4	0.1	2B
Chrysene	Chr	4	0.01	2B
Benzo[b]fluoranthene	BbF	5	0.1	2B
Benzo[k]fluoranthene	BkF	5	0.1	2B
Benzo[a]pyrene	BaP	5	1	1
Indeno[1,2,3-cd]pyrene	IcdP	6	0.1	2B
Dibenzo[a,h]anthracene	DahA	5	1	2A
Benzo[g,h,i]perylene	BghiP	6	0.01	3

**Table 3:** The mean concentration of PM_2.5_ and of PAHs (ng m^−3^) in charcoal barbecue restaurants in Chiang Mai City, Chiang Mai Province.

PAHs Compounds	Mean	Minimum	Maximum	S.D.
Naphthalene	36.20	33.06	39.75	2.14
Acenaphthylene	6.47	ND	25.93	7.92
Acenaphthene	2.95	ND	5.40	1.67
Fluorene	2.59	1.71	8.63	2.13
Phenanthrene	1.00	ND	6.75	2.26
Anthracene	0.68	0.48	1.04	0.18
Fluoranthene	2.41	ND	20.76	6.49
Pyrene	2.06	ND	12.26	3.82
Benzo (a) anthracene	15.66	9.92	27.35	5.59
Chrysene	15.73	2.56	32.56	11.74
Benzo (b) fluoranthene	11.70	8.57	15.67	2.22
Benzo (k) fluoranthene	8.44	ND	13.21	3.42
Benzo (a) pyrene	8.47	6.98	14.34	2.14
Indenol (1,2,3-cd) pyrene	7.12	ND	19.54	9.22
Dibenz (a,h) anthracene	2.27	ND	17.91	5.70
Benzo (g,h,i) perylene	8.98	ND	19.52	9.48
ncPAHs	63.34	41.45	109.16	22.46
cPAHs	69.40	35.39	123.47	32.31
∑PAHs	132.74	76.84	204.07	48.69
PM_2.5_ (µg m^−3^)	87.64	42.67	134.72	30.98

**Table 4:** The BaP toxic equivalent concentration (TEQ_BaP_) and the inhalation incremental lifetime cancer risk (ILCRinh) are presented for restaurant diners, categorized by exposure scenario. ILCR values are on the WHO unit-risk basis; the child receptor includes an ADAF of 3.

Scenario	Receptor	ED (y)	Mean	Median	Min	Max
TEQ_BaP_ (ng m^−3^)	—	—	15.34	12.76	9.90	33.51
Occasional (12 visits y^−1^)	Adults	24	1.88 × 10^−6^	1.56 × 10^−6^	1.21 × 10^−6^	4.11 × 10^−6^
Occasional (12 visits y^−1^)	Children	6	1.41 × 10^−6^	1.17 × 10^−6^	9.10 × 10^−7^	3.08 × 10^−6^
Regular (52 visits y^−1^)	Adults	24	8.15 × 10^−6^	6.78 × 10^−6^	5.26 × 10^−6^	1.78 × 10^−5^
Regular (52 visits y^−1^)	Children	6	6.11 × 10^−6^	5.08 × 10^−6^	3.94 × 10^−6^	1.34 × 10^−5^
Frequent (180 visits y^−1^)	Adults	24	2.82 × 10^−5^	2.35 × 10^−5^	1.82 × 10^−5^	6.16 × 10^−5^
Frequent (180 visits y^−1^)	Children	6	2.12 × 10^−5^	1.76 × 10^−5^	1.37 × 10^−5^	4.62 × 10^−5^
US EPA IRIS cross-check, frequent scenario	Adults	24	1.95 × 10^−7^	1.62 × 10^−7^	1.26 × 10^−7^	4.25 × 10^−7^
US EPA IRIS cross-check, frequent scenario	Children	6	1.46 × 10^−7^	1.21 × 10^−7^	9.42 × 10^−8^	3.19 × 10^−7^

## Data Availability

The data presented in this study are available on request from the corresponding author due to their sensitive nature.

## Supplementary Materials

The following supporting information can be downloaded at: https://www.mdpi.com/article/10.3390/toxics14070643/s1. Table S1: Characteristics of the ten sampled charcoal Moo Kratha restaurants (S1–S10) in Chiang Mai City, Thailand, with the corresponding PM2.5 and total PAH (ΣPAHs) concentrations measured in this study; Table S2: Concentrations of the 16 US EPA priority PM2.5-bound PAHs at each Moo Kratha restaurant (S1–S10), Chiang Mai City, Thailand. Concentrations are in ng m^−3^ unless otherwise stated; PM_2.5_ is in µg m^−3^. Each value is a single 3-hour sample collected during the peak evening service period (February 2026).

## References

[1] Brook R.D., Rajagopalan S., Pope C.A., Brook J.R., Bhatnagar A., Diez-Roux A.V., Holguin F., Hong Y., Luepker R.V., Mittleman M.A. (2010). Particulate matter air pollution and cardiovascular disease: An update to the scientific statement from the American Heart Association. Circulation.

[2] Pope C.A., Dockery D.W. (2006). Health Effects of Fine Particulate Air Pollution: Lines that Connect. J. Air Waste Manag. Assoc..

[3] Ponsawansong P., Prapamontol T., Rerkasem K., Chantara S., Tantrakarnapa K., Kawichai S., Li G., Fang C., Pan X., Zhang Y. (2023). Sources of PM_2.5_ Oxidative Potential during Haze and Non-haze Seasons in Chiang Mai, Thailand. Aerosol Air Qual. Res..

[4] Chi N.D.T., Huy D.H., Nguyen N.T., Nguyen L.S.P., Ngan T.A., Takenaka N., Hien T.T. (2024). PM_2.5_-bound Polycyclic Aromatic Hydrocarbons in Ho Chi Minh City, Vietnam: Potential Sources, Health Risks, and Deposition Flux. Aerosol Air Qual. Res..

[5] Siudek P. (2022). Seasonal distribution of PM_2.5_-bound polycyclic aromatic hydrocarbons as a critical indicator of air quality and health impact in a coastal-urban region of Poland. Sci. Total Environ..

[6] Kawichai S., Prapamontol T., Chantara S., Kanyanee T. (2020). Seasonal Variation and Sources Estimation of PM_2.5_-Bound PAHs from the Ambient Air of Chiang Mai City: An All-year-round Study in 2017. Chiang Mai J. Sci..

[7] Sekar M., T R P. (2024). Critical review on the formations and exposure of polycyclic aromatic hydrocarbons (PAHs) in the conventional hydrocarbon-based fuels: Prevention and control strategies. Chemosphere.

[8] Subair M.Y., Karigowda, Habib G., Warsi A.B., Imran M., Nabi M.U. (2026). Chemical investigation of polycyclic aromatic hydrocarbon sources and associated health risks in PM_2.5_ from Eastern India. Sci. Rep..

[9] Thepnuan D., Yabueng N., Chantara S., Prapamontol T., Tsai Y.I. (2020). Simultaneous determination of carcinogenic PAHs and levoglucosan bound to PM_2.5_ for assessment of health risk and pollution sources during a smoke haze period. Chemosphere.

[10] Kawichai S., Prapamontol T., Cao F., Song W., Zhang Y. (2022). Source Identification of PM_2.5_ during a Smoke Haze Period in Chiang Mai, Thailand, Using Stable Carbon and Nitrogen Isotopes. Atmosphere.

[11] Kawichai S., Prapamontol T., Cao F., Song W., Zhang Y.L. (2024). Characteristics of Carbonaceous Species of PM_2.5_ in Chiang Mai City, Thailand. Aerosol Air Qual. Res..

[12] Saksakulkrai S., Chantara S., Kraisitnitikul P., Srivastava D., Shi Z. (2026). Unveiling the origins of Northern Thailand’s haze: Comprehensive chemical characterization and source apportionment of PM_2.5_ using targeted molecular markers. J. Environ. Sci..

[13] Song W., Hong Y., Zhang Y., Cao F., Rauber M., Santijitpakdee T., Kawichai S., Prapamontol T., Szidat S., Zhang Y.L. (2024). Biomass Burning Greatly Enhances the Concentration of Fine Carbonaceous Aerosols at an Urban Area in Upper Northern Thailand: Evidence From the Radiocarbon-Based Source Apportionment on Size-Resolved Aerosols. J. Geophys. Res. Atmos..

[14] Song W., Zhang Y., Gao M., Xie F., Cao F., Rauber M., Kawichai S., Prapamontol T., Szidat S., Peng Y. (2026). Biomass burning increase in Southeast Asia is dominated by char black carbon. Commun. Earth Environ..

[15] Chotamonsak C., Thanadolmethaphorn P., Lapyai D., Chimla S. (2026). Near Real-Time Biomass Burning PM_2.5_ Emission Estimation to Support Environmental Health Risk Management in Northern Thailand Using FINNv2.5. Toxics.

[16] Kliengchuay W., Niampradit S., Sahanavin N., Mueller W., Todd S., Loh M., Johnston H., Vardoulakis S., Suwanmanee S., Phonphan W. (2025). Seasonal Analysis of Indoor and Outdoor Ratios of PM_2.5_ and PM_10_ in Bangkok and Chiang Mai: A Comparative Study of Haze and Non-Haze Episodes. Heliyon.

[17] Suriyawong P., Chuetor S., Samae H., Piriyakarnsakul S., Amin M., Furuuchi M., Hata M., Inerb M., Phairuang W. (2023). Airborne particulate matter from biomass burning in Thailand: Recent issues, challenges, and options. Heliyon.

[18] Thongsame W., Henze D.K., Barth M., Pfister G., Kumar R., Macatangay R., Hassan Bran S. (2025). Source Attribution and Health Burden of PM_2.5_ in Mainland Thailand. GeoHealth.

[19] Amnuaylojaroen T., Kaewkanchanawong P., Panpeng P. (2023). Distribution and Meteorological Control of PM_2.5_ and Its Effect on Visibility in Northern Thailand. Atmosphere.

[20] Sukkhum S., Lim A., Ingviya T., Saelim R. (2022). Seasonal Patterns and Trends of Air Pollution in the Upper Northern Thailand from 2004 to 2018. Aerosol Air Qual. Res..

[21] Callén M.S., Iturmendi A., López J.M. (2014). Source apportionment of atmospheric PM_2.5_-bound polycyclic aromatic hydrocarbons by a PMF receptor model. Assessment of potential risk for human health. Environ. Pollut..

[22] Dvorská A., Lammel G., Klánová J. (2011). Use of diagnostic ratios for studying source apportionment and reactivity of ambient polycyclic aromatic hydrocarbons over Central Europe. Atmos. Environ..

[23] Qu L., Yang L., Zhang Y., Wang X., Sun R., Li B., Lv X., Chen Y., Wang Q., Tian C. (2022). Source Apportionment and Toxic Potency of PM_2.5_-Bound Polycyclic Aromatic Hydrocarbons (PAHs) at an Island in the Middle of Bohai Sea, China. Atmosphere.

[24] Palazzi P., Mezzache S., Bourokba N., Hardy E.M., Schritz A., Bastien P., Emond C., Li J., Soeur J., Appenzeller B.M.R. (2018). Exposure to polycyclic aromatic hydrocarbons in women living in the Chinese cities of BaoDing and Dalian revealed by hair analysis. Environ. Int..

[25] Radarit K., Wiriya W., Chai-Adisaksopha C., Chantara S., Prapamontol T. (2024). Significantly Increased Accumulations of PAHs in Scalp Hair During Smoke-haze Period Among Female Adolescents in Chiang Mai, Thailand. Nat. Life Sci. Commun..

[26] Shiyi Z., Weikeng L., Zhao F., Huang L., Qin R., Yan X., Tang B., Luo X., Mai B., Yu Y. (2024). Melanin-Mediated Accumulation of Polycyclic Aromatic Hydrocarbons in Human Hair: Insights from Biomonitoring and Cell Exposure Studies. J. Hazard. Mater..

[27] Kaltsonoudis C., Kostenidou E., Louvaris E., Psichoudaki M., Tsiligiannis E., Florou K., Liangou A., Pandis S.N. (2017). Characterization of fresh and aged organic aerosol emissions from meat charbroiling. Atmos. Chem. Phys..

[28] Klein F., Baltensperger U., Prévôt A.S.H., El Haddad I. (2019). Quantification of the impact of cooking processes on indoor concentrations of volatile organic species and primary and secondary organic aerosols. Indoor Air.

[29] Tang R., Sahu R., Su Y., Milsom A., Mishra A., Berkemeier T., Pfrang C. (2024). Impact of Cooking Methods on Indoor Air Quality: A Comparative Study of Particulate Matter (PM) and Volatile Organic Compound (VOC) Emissions. Indoor Air.

[30] Li N., Bhattacharya P., Karavalakis G., Williams K., Gysel N., Rivera-Rios N. (2014). Emissions from commercial-grade charbroiling meat operations induce oxidative stress and inflammatory responses in human bronchial epithelial cells. Toxicol. Rep..

[31] Mencarelli A., Greco R., Balzan S., Grigolato S., Cavalli R. (2023). Charcoal-based products combustion: Emission profiles, health exposure, and mitigation strategies. Environ. Adv..

[32] Kim S.C., Lee T.J., Jeon J.M., Kim D.S., Jo Y.M. (2020). Emission Characteristics and Control Device Effectiveness of Particulate Matters and Particulate-phase PAHs from Urban Charbroiling Restaurants: A Field Test. Aerosol Air Qual. Res..

[33] Wei See S., Karthikeyan S., Balasubramanian R. (2006). Health risk assessment of occupational exposure to particulate-phase polycyclic aromatic hydrocarbons associated with Chinese, Malay and Indian cooking. J. Environ. Monit..

[34] Sharma D., Jain S. (2020). Carcinogenic risk from exposure to PM_2.5_ bound polycyclic aromatic hydrocarbons in rural settings. Ecotoxicol. Environ. Saf..

[35] Wu M.T., Lin P.C., Pan C.H., Peng C.Y. (2019). Risk assessment of personal exposure to polycyclic aromatic hydrocarbons and aldehydes in three commercial cooking workplaces. Sci. Rep..

[36] Zhang J.F., Chen X., Gao K., Cheng S.Y., Duan W.J., Fu L.Y., Li J.J., Lan S.S., Fang C.L. (2025). Health Risks from Exposure to PM_2.5_-bound Polycyclic Aromatic Hydrocarbons in Fumes Emitted from Various Cooking Styles and Their Respiratory Deposition in a City Population Stratified by Age and Sex. Biomed. Environ. Sci..

[37] ChooChuay C., Pongpiachan S., Tipmanee D., Deelaman W., Iadtem N., Suttinun O., Wang Q., Xing X., Loi G., Han Y. (2022). Effects of Agricultural Waste Burning on PM_2.5_-Bound Polycyclic Aromatic Hydrocarbons, Carbonaceous Compositions, and Water-Soluble Ionic Species in the Ambient Air of Chiang-Mai, Thailand. Polycycl. Aromat. Compd..

[38] Janta R., Sekiguchi K., Yamaguchi R., Sopajaree K., Pongpiachan S., Chetiyanukornkul T. (2019). Ambient PM_2.5_, Polycyclic Aromatic Hydrocarbons and Biomass Burning Tracer in Mae Sot District, Western Thailand. Atmos. Pollut. Res..

[39] Pongpiachan S., Hattayanon M., Cao J. (2017). Effect of agricultural waste burning season on PM_2.5_-bound polycyclic aromatic hydrocarbon (PAH) levels in Northern Thailand. Atmos. Pollut. Res..

[40] (1984). Method 610: Determination of Polynuclear Aromatic Hydrocarbons (16 Priority Pollutants).

[41] Alomirah H., Al-Zenki S., Al-Hooti S., Zaghloul S., Sawaya W., Ahmed N., Kannan K. (2011). Concentrations and dietary exposure to polycyclic aromatic hydrocarbons (PAHs) from grilled and smoked foods. Food Control.

[42] Yang T.T., Hsu C.Y., Chen Y.C., Young L.H., Huang C.H., Ku C.H. (2017). Characteristics, Sources, and Health Risks of Atmospheric PM_2.5_-Bound Polycyclic Aromatic Hydrocarbons in Hsinchu, Taiwan. Aerosol Air Qual. Res..

[43] Si J., Bai L., Xu X., Li C. (2023). Pollution characteristics and health hazards of PAHs in PM_1.0_ in the cooking environment. Build. Environ..

[44] Manoli E., Kouras A., Karagkiozidou O., Argyropoulos G., Voutsa D., Samara C. (2016). Polycyclic aromatic hydrocarbons (PAHs) at traffic and urban background sites of northern Greece: Source apportionment of ambient PAH levels and PAH-induced lung cancer risk. Environ. Sci. Pollut. Res. Int..

[45] Nisbet I.C.T., LaGoy P.K. (1992). Toxic equivalency factors (TEFs) for polycyclic aromatic hydrocarbons (PAHs). Regul. Toxicol. Pharmacol..

[46] World Health Organization (2010). WHO Guidelines for Indoor Air Quality: Selected Pollutants.

[47] U.S. EPA (2005). Supplemental Guidance for Assessing Susceptibility from Early-Life Exposure to Carcinogens.

[48] U.S. EPA (2017). Integrated Risk Information System (IRIS) Chemical Assessment Summary: Benzo[a]pyrene (CASRN 50-32-8).

[49] U.S. EPA (2011). Exposure Factors Handbook: 2011, Edition.

[50] Goudarzi G., Baboli Z., Moslemnia M., Tobekhak M., Tahmasebi Birgani Y., Neisi A., Ghanemi K., Babaei A.A., Hashemzadeh B., Ahmadi Angali K. (2021). Assessment of incremental lifetime cancer risks of ambient air PM_10_-bound PAHs in oil-rich cities of Iran. J. Environ. Health Sci. Eng..

[51] Zhang X., Lu W., Xu L., Wu W., Sun B., Fan W., Zheng H., Huang J. (2022). Environmental Risk Assessment of Polycyclic Aromatic Hydrocarbons in Farmland Soils near Highways: A Case Study of Guangzhou, China. Int. J. Environ. Res. Public Health.

[52] Mohamadian Geravand P., Goudarzi G., Ahmadi M. (2024). Polycyclic aromatic hydrocarbons (PAHs) in urban street dust in Masjed Soleyman, Khuzestan, Iran: Sources and health risk assessment. Int. J. Environ. Anal. Chem..

[53] Room S.A., Lin C.E., Pan S.Y., Hsiao T.C., Chou C.C.K., Chi K.H. (2023). Incremental Lifetime Cancer Risk of PAHs in PM2.5 via Local Emissions and Long-Range Transport during Winter. Aerosol Air Qual. Res..

[54] Wei Y., Han I.K., Hu M., Shao M., Zhang J.J., Tang X. (2010). Personal exposure to particulate PAHs and anthraquinone and oxidative DNA damages in humans. Chemosphere.

[55] Wang W., Huang M.J., Kang Y., Wang H.S., Leung A.O., Cheung K.C., Wong M.H. (2011). Polycyclic aromatic hydrocarbons (PAHs) in urban surface dust of Guangzhou, China: Status, sources and human health risk assessment. Sci. Total Environ..

[56] Vanphanom S., Viengnakhone V., Kongmany C., Souksamone T., Bounmany S., Diane A., Connie O.N., Jo D. (2023). Grill workers and air pollution health effects from charcoal combustion in Vientiane capital. J. Air Pollut. Health.

[57] Conchione C., Socal S., Barp L., Moret S. (2025). Evaluation of Polycyclic Aromatic Hydrocarbons (PAHs) in Pork Meat Cooked with Two Different Methods. Molecules.

[58] Balducci C., Santoro S., Bencardino M., D’Amore F., Cerasa M., Formenton G., Leonardi C. (2026). Evaluation of the Role of Benzo(a)pyrene as Carcinogenic Index of PM_10_-Bound PAHs in Italian Urban Sites. Environments.

[59] Badyda A.J., Rogula-Kozłowska W., Majewski G., Bralewska K., Widziewicz-Rzońca K., Piekarska B., Rogulski M., Bihałowicz J.S. (2022). Inhalation risk to PAHs and BTEX during barbecuing: The role of fuel/food type and route of exposure. J. Hazard. Mater..

[60] Oliveira M., Capelas S., Delerue-Matos C., Morais S. (2020). Grill Workers Exposure to Polycyclic Aromatic Hydrocarbons: Levels and Excretion Profiles of the Urinary Biomarkers. Int. J. Environ. Res. Public Health.

