# Supplementary material for: Characteristics and Carcinogenic Risk of PM2.5-Bound Polycyclic Aromatic Hydrocarbons from Charcoal Barbecue (Moo Kratha) Restaurants in Chiang Mai, Thailand
Source: Toxics. 2026 Jul 22;14(7):643. doi: 10.3390/toxics14070643 (PMC13419146; doi:10.3390/toxics14070643)
Supplement: Supplementary file 1 [file toxics-14-00643-s001.zip › toxics-4405647-supplementary.pdf]

Characteristics and Carcinogenic Risk of $PM_{2.5}$-bound Polycyclic Aromatic Hydrocarbons from Charcoal Barbecue (Moo Kratha) Restaurants in Chiang Mai, Thailand

Thanawat Komonnithiphong[1,2], Kotchakorn Khammoon[1], Sakaewan Ounjaijean[2], Kongsak Boonyapranai[2], Hataichanok Chuljerm[2], Kanokwan Kulprachakarn[2], Wiritphon Khiaolaongam[2], Anurak Wongta[2], Surat Hongsibsong[2], Sawaeng Kawichai[1]*

[1] Research Institute for Health Sciences (RIHES), Chiang Mai University, Chiang Mai, Thailand; sawaeng.kaw@cmu.ac.th (S.K.); thanawat_komo@cmu.ac.th (T.K.); kotchakorn_kh@cmu.ac.th (K.Kh.)

[2] School of Health Sciences Research, Research Institute for Health Sciences, Chiang Mai University, Chiang Mai 50200, Thailand; sakaewan.o@cmu.ac.th (S.O.); kongsak.b@cmu.ac.th (K.B.); hataichanok.ch@cmu.ac.th (H.C.); kanokwan.kul@cmu.ac.th (K.Ku.); wiritphon_k@cmu.ac.th (W.K.); anurak.wongta@cmu.ac.th (A.W.); surat.hongsibsong@cmu.ac.th (S.H.)

* Correspondence: sawaeng.kaw@cmu.ac.th (S.K.)

Table S1. Characteristics of the ten sampled charcoal Moo Kratha restaurants (S1–S10) in Chiang Mai City, Thailand, with the corresponding $PM_{2.5}$ and total PAH (ΣPAHs) concentrations measured in this study.

Site	Floor area (m^2)	Seating capacity (persons)	Ventilation type [a]	Seating layout [b]	Distance to nearest major road (m)	$PM_{2.5}$ ($\mu g\ m^{-3}$) [c]	ΣPAHs ($ng\ m^{-3}$) [c]
**S1**	180	80	Semi-enclosed	Indoor/Outdoor	45	78.47	95.20
**S2**	320	180	Semi-enclosed	Indoor/Outdoor	20	127.84	204.07
**S3**	300	160	Semi-enclosed	Indoor/Outdoor	30	134.72	204.06
**S4**	150	60	Open-air	Indoor/Outdoor	80	73.35	90.43
**S5**	170	70	Open-air	Indoor/Outdoor	70	78.95	98.48
**S6**	260	140	Semi-enclosed	Indoor/Outdoor	35	94.75	148.64
**S7**	170	70	Open-air	Indoor/Outdoor	75	43.75	89.58
**S8**	160	60	Open-air	Indoor/Outdoor	90	42.67	76.84
**S9**	280	159	Semi-enclosed	Indoor/Outdoor	25	107.64	165.43
**S10**	250	130	Semi-enclosed	Indoor/Outdoor	40	94.33	154.69

[a] Ventilation type: open-air (no enclosure), semi-enclosed (partial walls and/or roof), or mechanically ventilated (extraction hood or forced-air exhaust over the grilling area).
[b] Seating layout: indoor, outdoor, or mixed (both indoor and outdoor seating).
[c] $PM_{2.5}$ and ΣPAHs values are taken from this study.

Table S2. Concentrations of the 16 US EPA priority $PM_{2.5}$-bound PAHs at each Moo Kratha restaurant (S1–S10), Chiang Mai City, Thailand. Concentrations are in ng m^{-3} unless otherwise stated; $PM_{2.5}$ is in μg m^{-3}. Each value is a single 3-hour sample collected during the peak evening service period (February 2026).

**PAH compounds**	**S1**	**S2**	**S3**	**S4**	**S5**	**S6**	**S7**	**S8**	**S9**	**S10**	**Mean ± SD**
Naphthalene (Nap)	38.94	35.59	39.75	37.20	35.51	36.20	36.99	35.34	33.06	33.37	36.20±2.14
Acenaphthylene (Acy)	7.20	8.37	6.53	ND	ND	25.93	6.49	ND	ND	10.17	6.47±7.92
Acenaphthene (Ace)	3.46	5.40	3.45	3.44	3.47	3.40	3.43	3.46	ND	ND	2.95±1.67
Fluorene (Flu)	1.82	2.11	1.71	8.63	2.03	1.77	1.95	2.16	1.96	1.79	2.59±2.13
Phenanthrene (Phe)	ND	3.23	6.75	ND	ND	ND	ND	ND	ND	ND	1.00±2.26
Anthracene (Ant)	0.62	1.04	0.85	0.48	0.69	0.78	0.53	0.50	0.71	0.60	0.68±0.18
Fluoranthene (Fla)	ND	2.05	20.76	ND	ND	ND	ND	ND	ND	1.29	2.41±6.49
Pyrene (Pyr)	ND	3.29	12.26	ND	ND	1.49	ND	ND	0.40	3.21	2.06±3.82
Benzo(a)anthracene (BaA)	10.58	21.22	17.24	10.53	14.74	16.27	11.12	9.92	27.35	17.64	15.66±5.59
Chrysene (Chr)	5.80	32.56	28.53	4.15	10.12	16.05	5.06	2.56	29.46	23.01	15.73±11.74
Benzo(b)fluoranthene (BbF)	10.61	14.15	11.85	10.40	12.36	12.30	8.76	8.57	15.67	12.37	11.70±2.22
Benzo(k)fluoranthene (BkF)	8.89	9.52	7.94	7.41	11.15	8.57	8.27	ND	13.21	9.47	8.44±3.42
Benzo(a)pyrene (BaP)	7.27	8.56	8.00	8.19	8.40	8.04	6.98	14.34	7.93	7.01	8.47±2.14
Indeno(1,2,3-cd)pyrene (IcdP)	ND	19.54	16.55	ND	ND	ND	ND	ND	17.81	17.29	7.12±9.22
Dibenz(a,h)anthracene (DahA)	ND	17.91	4.78	ND	ND	ND	ND	ND	ND	ND	2.27±5.70
Benzo(g,h,i)perylene (BghiP)	ND	19.52	17.11	ND	ND	17.83	ND	ND	17.86	17.47	8.98±9.48
**ncPAHs**	52.04	80.61	109.16	49.76	41.70	87.41	49.39	41.45	53.99	67.91	63.34±22.46
**cPAHs**	43.15	123.47	94.89	40.67	56.78	61.23	40.19	35.39	111.43	86.79	69.40±32.31
**∑PAHs**	95.20	204.07	204.06	90.43	98.48	148.64	89.58	76.84	165.43	154.69	132.74±48.69
**$PM_{2.5}$ (μg m^{-3})**	78.47	127.84	134.72	73.35	78.95	94.75	43.75	42.67	107.64	94.33	87.64±30.98

Abbreviations: ND, not detected; ncPAHs, non-carcinogenic PAHs; cPAHs, carcinogenic PAHs (the sum of BaA, Chr, BbF, BkF, BaP, DahA, and IcdP); ∑PAHs, sum of the 16 PAHs. Means and standard deviations were calculated across all ten sites with non-detected values treated as zero, consistent with Table 3.
